# The economic impact of comorbidity in multiple sclerosis

**DOI:** 10.1007/s10072-022-06517-7

**Published:** 2022-11-28

**Authors:** Michela Ponzio, Maria Cristina Monti, Giulia Mallucci, Paola Borrelli, Sara Fusco, Andrea Tacchino, Giampaolo Brichetto, Livio Tronconi, Cristina Montomoli, Roberto Bergamaschi

**Affiliations:** 1 Scientific Research Area, Italian Multiple Sclerosis Foundation (FISM), Genoa, Italy; 2 Department of Public Health, Experimental and Forensic Medicine, Unit of Biostatistics and Clinical Epidemiology, University of Pavia, Pavia, Italy; 3 Multiple Sclerosis Center, IRCCS Mondino Foundation, Pavia, Italy; 4 Department of Medical, Oral and Biotechnological Sciences, Laboratory of Biostatistics, University ’’G. d’Annunzio’’ Chieti-Pescara, Chieti, Italy; 5 AISM Rehabilitation Service, Italian Multiple Sclerosis Society, Genoa, Italy; 6 Legal Medicine Unit, IRCCS Mondino Foundation, Pavia, Italy; 7 Department of Public Health, Experimental and Forensic Medicine, Forensic Science Unit, University of Pavia, Pavia, Italy

**Keywords:** Comorbidity, Multiple sclerosis, Economic burden, Costs

## Abstract

**Background:**

Comorbid conditions are common in people with multiple sclerosis (pwMS). They can delay diagnosis and negatively impact the disease course, progression of disability, therapeutic management, and adherence to treatment.

**Objective:**

To quantify the economic impact of comorbidity in multiple sclerosis (MS), based on cost-of-illness estimates made using a bottom-up approach.

**Methods:**

A retrospective study was carried out in two northern Italian areas. The socio-demographic and clinical information, including comorbidities data, were collected through ad hoc anonymous self-assessment questionnaire while disease costs (direct and indirect costs of disease and loss of productivity) were estimated using a bottom-up approach. Costs were compared between pwMS with and without comorbidity. Adjusted incremental costs associated with comorbidity were reported using generalized linear models with log-link and gamma distributions or two-part models.

**Results:**

51.0% of pwMS had at least one comorbid condition. Hypertension (21.0%), depression (15.7%), and anxiety (11.7%) were the most prevalent. PwMS with comorbidity were more likely to use healthcare resources, such as hospitalizations (OR = 1.21, *p* < 0.001), tests (OR = 1.59, *p* < 0.001), and symptomatic drugs and supplements (OR = 1.89, *p* = 0.012), and to incur non-healthcare costs related to investment (OR = 1.32, *p* < 0.001), transportation (OR = 1.33, *p* < 0.001), services (OR = 1.33, *p* < 0.001), and informal care (OR = 1.43, *p* = 0.16). Finally, they experienced greater productivity losses (OR = 1.34, *p* < 0.001) than pwMS without comorbidity. The adjusted incremental annual cost per patient due to comorbidity was €3,106.9 (13% of the overall costs) with MS disability found to exponentially affect annual costs.

**Conclusion:**

Comorbidity has health, social, and economic consequences for pwMS.

## Introduction

Comorbidity is the coexistence of one of more chronic conditions with an index disease of interest. Many individuals with a chronic disease suffer from a coexisting (comorbid) chronic condition, and the likelihood of comorbidity increases with age [1]. In patients over 65 years of age, additional chronic conditions have been found to exponentially increase healthcare costs, suggesting that some of the added costs are generated by medical complexity and/or inefficient treatment of comorbid conditions. [2, 3] Comorbidity in people with multiple sclerosis (pwMS) is an area attracting increasing interest. Comorbidity has been associated with delayed diagnosis of multiple sclerosis (MS), progression of MS disability, lower health-related quality of life in MS patients, increased MS burden on magnetic resonance imaging (MRI), and increased disease-related mortality [4–6, 7]. In recent decades, the MS population has changed. In line with longer life expectancies generally, and also thanks to the use of the more effective disease-modifying treatments (DMTs) [8], pwMS are now older, and therefore at higher risk of comorbidity. Compared with their peers in the general population, pwMS display increased rates of comorbidity, [9] with associated conditions including both mental (e.g., depression and anxiety) and somatic (e.g., hypertension, diabetes) diseases. [10]

The healthcare and social impacts of MS are high. In Italy, the disease has an estimated annual overall cost of €4.8 billion [11]. Both direct costs, generated by healthcare resource consumption, and indirect costs, such as those associated with informal care, services, and loss of productivity, are high. These direct and indirect costs reflect the disability status of MS patients, higher costs being linked to increased disability. [12, 13]

Although the economic cost of MS has been examined in the literature, [14] evidence on the specific contribution of comorbidity in this setting is lacking. The purpose of this study was to quantify the economic impact of comorbidity in MS, considering all types of cost (healthcare- and non-healthcare-related expenditure and lost productivity).

## Methods

### Study design and population

The study was a retrospective multicenter study aiming to estimate the economic impact of comorbidity in a sample of MS patients in 2020.

Subjects were enrolled at the MS Center of the Mondino Foundation, Pavia, and at the AISM (Italian Multiple Sclerosis Association) Rehabilitation Service in Liguria, Genoa. Inclusion criteria were as follows: a diagnosis of MS, [15] age ≥ 18 years, and being a patient registered within the administrative areas of Pavia or Genoa. Before entering the study, all enrolled participants gave their written informed consent in accordance with the revised Declaration of Helsinki.

### Data collection

Participants completed an ad hoc anonymous self-assessment questionnaire, which collected socio-demographic data and clinical information on MS, including disease-related disability measured using the self-assessed Expanded Disability Status Scale (EDSS) [16]. The questionnaire also investigated the presence of the main comorbid conditions in MS, [10, 17] namely, depression, anxiety, hypertension, diabetes, hyperlipidemia, chronic lung disease, autoimmune diseases, cancer, heart disease, and vascular disease.

Patients who reported one or more comorbid condition were classified as “pwMS with comorbidity.”

Disease costs (direct and indirect costs of disease and loss of productivity in MS patients) were estimated using a bottom-up approach. Data on resource use were collected through a standardized questionnaire used in previous studies [13, 18]. To minimize recall bias, these data were collected retrospectively considering timeframes appropriate to each type of resource. Costs related to resource use, a function of the quantity of resources used and their cost, were calculated from a societal perspective, including all costs regardless of payers. The unit costs were derived from regional and national tariffs applied by the Italian national health service to reimburse providers, or directly from institutions or experts, while out-of-pocket and third-party expenditures were mostly reported directly by the patients. All costs were estimated in euros (€). To obtain the overall resource use cost per patient per year, the resource use data collected were annualized. The cost of informal care was calculated on the basis of leisure or working time lost by caregivers in order to provide care [19]. The human capital approach was used to estimate productivity losses both of patients, and of caregivers due to work time lost in order to care for patients. [20]

Costs were categorized into healthcare costs, non-healthcare costs, and patients’ productivity losses.

Healthcare costs referred to hospitalizations (inpatient stays or day admissions), specialist consultations (with specialists or general practitioners), consultations with healthcare professionals other than neurologists and GPs (nurses, physiotherapists, psychologists), tests (instrumental examinations such as MRI scans, evoked potentials, computed tomography, ultrasound, electrocardiogram, blood tests, urine tests, X-ray, echo-Doppler tests, myography, and other tests), DMTs, and symptomatic drugs and supplements.

The non-healthcare costs considered were defined as followed: investments (home and car adaptations due to MS), aids, transportation (for health reasons), services (personal assistant and home help), and informal care. Productivity losses (short-term reduction and loss of working activity and loss of work days) were calculated in patients < 65 years of age and in patients of working age who had taken early retirement due to MS.

### Statistical analysis

For our analysis, we used annual expenditure outcomes per person (healthcare and non-healthcare expenditure, productivity loss, and total expenditure) as well as separate annual expenditure per person per item of expenditure (inpatient stay or day admission, specialist consultations, consultations with other healthcare professionals, tests, drug use, investments, aids, transportation, services, and informal care). Continuous measures were summarized as mean and standard deviation (SD) or median and interquartile range (IQR). Categorical measures were summarized as counts and percentages. General demographic questions were used to describe the study sample and to allow for subgroup analyses of costs.

Normality distribution for quantitative variables was assessed using the Shapiro–Wilk test. Pearson’s chi-square tests for categorical variables and Student’s *t*‐test for independent data, or the non-parametric Wilcoxon rank-sum test when appropriate, were used to compare the characteristics of the “pwMS without comorbidity” and “pwMS with comorbidity” groups.

The probability of resource use, in relation to the main healthcare and non-healthcare items, was estimated in and compared between the two groups. Utilization of specific resource types (as the independent variable) was categorized as “no resources used” (taken as the reference category) and “at least one resource used.” Unadjusted and adjusted odds ratios (ORs) between the two comorbidity groups were calculated with their respective 95% confidence intervals (CIs) and *p*-values. Adjusted ratios were estimated using multivariate logistic regression models, controlling for age, sex, educational level, and disability level (EDSS score).

Unadjusted and adjusted cost differences for main expenditure outcomes and for each item of expenditure between the two comorbidity groups were analyzed using generalized linear models (GLMs) with log-link and gamma distributions or two-part models for items with more than 5% zero values. These latter models are appropriate for analyzing zero-inflated cost data with skewness, [21] which is typical in medical expenditure data [22]. The model was composed of a logistic regression for the probability of observing positive-versus-zero expenditure outcome, followed by a GLM with log-link and gamma distribution, fitted for those participants showing non-zero expenditure outcome. To improve the interpretation of the coefficients from the two-part models, we generated a marginal (or incremental) effect of each variable on expenditure outcome [23]. Adjusted cost differences were estimated, controlling for age, sex, and disability level.

For all models, standard errors allowing for intragroup correlation were calculated (using a clustered sandwich estimator of the variance) to take account of the nested effect of living in two geographical areas (i.e., Pavia and Genoa, where the participating MS centers are located). The unadjusted and adjusted cost differences were also calculated for comorbid conditions that presented a frequency > 5% (category reference: no comorbidity). The level of significance was set at *p* < 0.05. The analyses were performed using Stata Statistical Software (StataCorp, 2017).

## Results

### *Sample characteristics (N* = *600)*

**Fig. 1:**
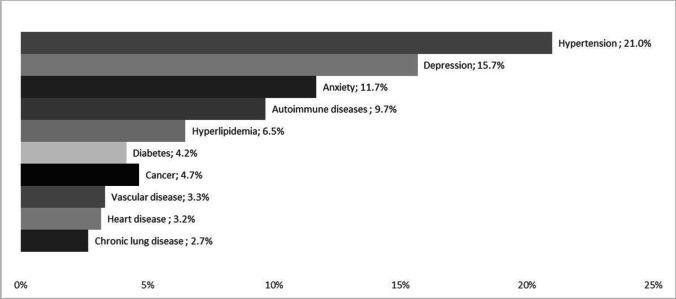
Comorbidity prevalence rates

We enrolled 600 pwMS, of whom 306 (51.0%) reported comorbidity. One comorbid condition was reported in 168 (28.0%) pwMS, two comorbid conditions in 97 (16.2%), and three or more in the remaining 41 (6.8%). The five most reported comorbidities were hypertension (21.0%), depression (15.7%), anxiety (11.7%), autoimmune diseases (9.7%), and hyperlipidemia (6.5%) (Fig. 1). PwMS with comorbidity were older (55.4 vs. 48.8 years, *p* < 0.001) and displayed higher MS disability compared with pwMS without comorbidity (median EDSS score: 3.5, IQR: 2.0–6.0 vs. median EDSS: 2.5, IQR 1.5–5.5; *p* = 0.007). Additionally, the group of pwMS with comorbidity had a higher percentage of women (68.6% vs. 60.2%, *p* = 0.031) and a lower educational level (up to primary school 37.3% vs. 28.4%, high school 44.9% vs 47.6%, and university 17.8% vs. 24.0%, *p* = 0.039), and a lower percentage of currently employed individuals (49.0% vs. 57.8%, *p* = 0.031) (Table 1).

**Table 1:** Socio-demographic and clinical characteristics of the sample by presence/absence of at least one comorbidity (*n* = 600)

		Without comorbidity *n* = 294	With comorbidity *n* = 306	*p* value
**Age** (years), mean (SD) Median (IQR)		48.8 (12.2) 49.0 (40.0–56.0)	55.4 (11.2) 56.0 (49.0–62.0)	< 0.001
**Sex,** *n* (%)	Male	117 (39.8%)	96 (31.4%)	0.031
	Female	177 (60.2%)	210 (68.6%)	
**Education,** *n* (%) *Missing data (0.8%)*	Primary school	83 (28.4%) 139 (47.6%) 70 (24.0%)	113 (37.3%) 136 (44.9%) 54 (17.8%)	0.039
	High school University degree			
**Currently employed,** *n* (%)		170 (57.8%)	150 (49.0%)	0.031
**Duration of illness,** mean (SD) Median (iQR) *Missing data (0.5%)*		16.4 (10.7) 14.0 (8.0–23.0)	17.9 (10.8) 16.5 (9.0–25.0)	0.067
**Type of MS,** *n* (%)	RR	216 (73.5%)	209 (68.7%)	0.421
	SP	60 (20.4%)	71 (23.4%)	
	PP	18 (6.1%)	24 (7.9%)	
**EDSS score,** mean (SD) Median (IQR)		3.3 (2.3) 2.5 (1.5–5.5)	3.8 (2.1) 3.5 (2.0–6.0)	0.007
**Relapses during last 3 months**, *n* (%)	Yes No Unsure	16 (5.4%) 275 (93.5%) 3 (1.0%)	12 (3.9%) 289 (94.4%) 5 (1.6%)	0.554

*SD*, standard deviation; *IQR*, interquartile range; *RR*, relapsing–remitting; *SP*, secondary-progressive; *PP*, primary-progressive; *EDSS*, Expanded Disability Status Scale

### Healthcare and non-healthcare resource utilization and lost productivity

PwMS with comorbidity used more healthcare and non-healthcare resources than pwMS without comorbidity (Table 2). Subjects with comorbidity were more likely to have hospitalizations (OR = 1.21, *p* < 0.001), undergo tests (OR = 1.59, *p* < 0.001), and use symptomatic drugs and supplements (OR = 1.89, *p* = 0.012). When running the unadjusted model, we observed a lower likelihood of DMT use in subjects with comorbidity (OR = 0.59, *p* = 0.003), but the statistical significance was not maintained in the adjusted model (OR = 0.90, *p* = 0.629).

**Table 2:** Estimate of risk of resource utilization among pwMS with comorbidity with respect to pwMS without comorbidity

	Unadjusted		*Adjusted	
	OR (95% IC)	*p* value	OR (95% IC)	*p* value
**Healthcare resources**				
Hospitalizations	1.08 (0.70–1.68)	0.729	1.21 (1.19–1.22)	< 0.001
Specialist consultations	1.13 (0.73–1.77)	0.583	1.19 (0.98–1.43)	0.076
Other health professional consultations	1.50 (1.09–2.07)	0.014	1.40 (0.89–2.20)	0.140
Tests	1.48 (1.04–2.09)	0.029	1.59 (1.45–1.73)	< 0.001
Use of symptomatic drugs and supplements	1.99 (1.39–2.84)	< 0.001	1.89 (1.15–3.11)	0.012
Disease-modifying drugs	0.59 (0.42–0.84)	0.003	0.90 (0.57–1.40)	0.629
**Non-healthcare resources**				
°Investments	1.68 (1.13–2.48)	0.010	1.32 (1.17–1.50)	< 0.001
Transportation	1.23 (0.89–1.70)	0.208	1.33 (1.17–1.52)	< 0.001
Services	1.89 (1.12–3.20)	0.018	1.52 (1.03–2.25)	0.034
Informal care	1.70 (1.21–2.39)	0.002	1.43 (1.07–1.92)	0.016

°Investments included equipment, aids, and modifications. The logistic regression models using the clustered sandwich estimator of the variance to take account of the nested geographical effect (Pavia and Genoa) where the MS centers are located. The reference category of the dependent variable was pwMS without comorbidity. *OR*, odds ratio; *CI*, confidence interval of the OR. *OR adjusted for age, sex, education level, and EDSS score

The pwMS with comorbidity were also more likely to incur costs related to investments (OR = 1.32, *p* < 0.001), transportation (OR = 1.33, *p* < 0.001), services (OR = 1.52, *p* = 0.034), and informal care (OR = 1.43, *p* = 0.16), also after controlling for potentially confounding factors. Moreover, pwMS with comorbidity more frequently experienced productivity losses than those without comorbidity (OR = 1.34, *p* < 0.001).

### Average and incremental costs of comorbidity

**Fig. 2:**
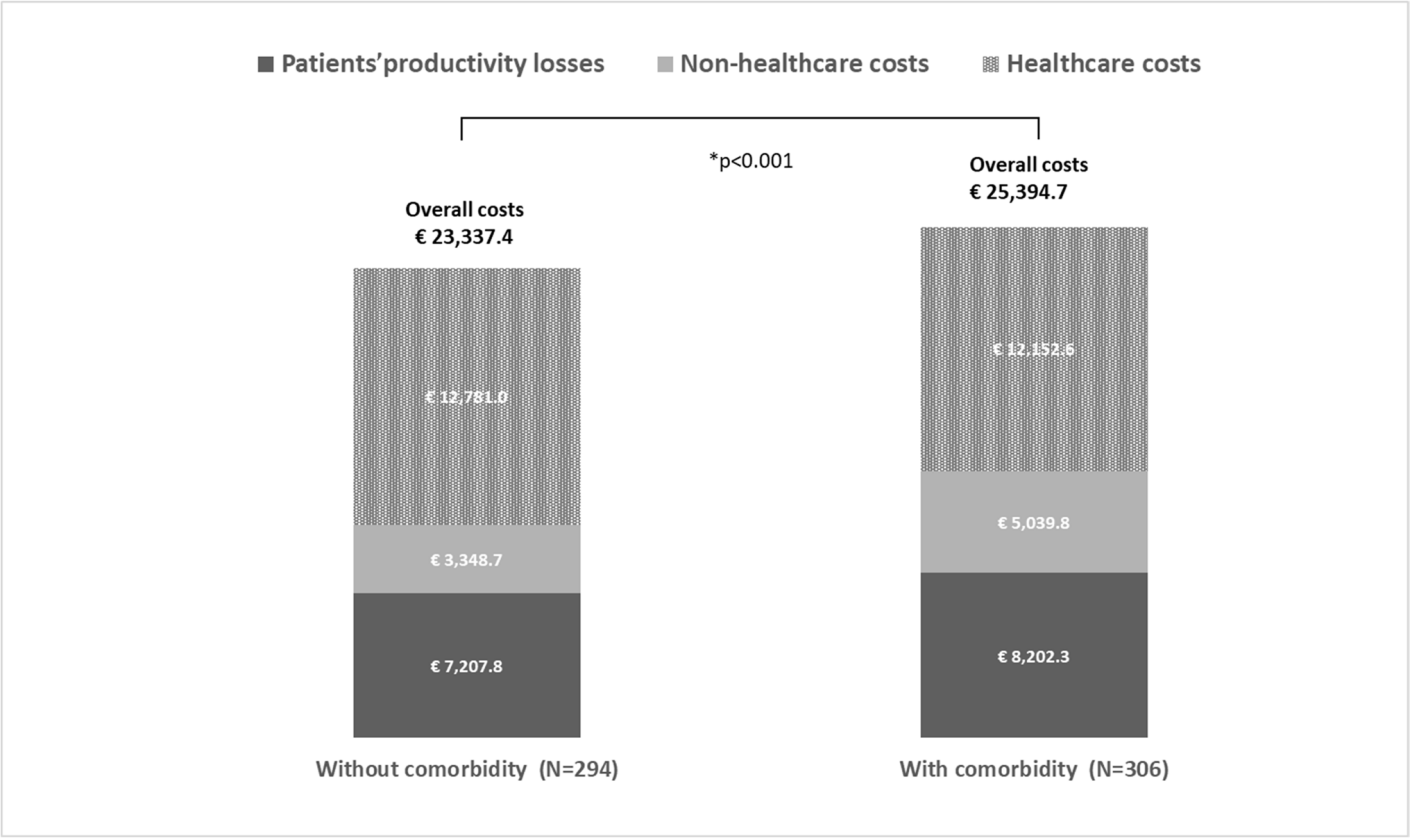
Mean annual costs per patient by presence/absence of comorbidity (2020). *The *p* value refers to adjusted (age, sex, education level, and EDSS score) overall cost differences between two comorbidity groups obtained using GLMs with log-link and gamma distributions. A clustered sandwich estimator of the variance was used to take account of the nested effect of living in two geographical regions (i.e., Pavia and Genoa, where the participating MS centers are located)

The annual overall costs incurred per patient amounted to €25,394.7 in the group with comorbidity and €23,337.4 in the group without comorbidity, and accordingly the total adjusted for age, sex, educational level, and MS disability (< 0.001) was also higher in pwMS with comorbidity (the mean adjusted incremental cost was €3,106.9). Figure 2 breaks the costs down into the three main categories: healthcare, non-healthcare, and lost productivity. The between-groups difference in the mean adjusted incremental cost was found to be significant both for healthcare costs and patients’ lost productivity (€873.2, *p* = 0.003 and €333.4, *p* = 0.046, respectively) (Table 3).

**Table 3:** Mean (SD) annual costs incremental costs of comorbidity in multiple sclerosis (2020, €)

	Without comorbidity *N* = 294	With comorbidity *N* = 306	Unadjusted incremental cost (A–B)	*Adjusted incremental cost (A–B)
	Average costs (SD) (A)	Average costs (SD) (B)	Coef (*p* value)	Coef (*p* value)
***Healthcare costs***	**12781.0 (12496.4)**	**12152.6 (14166.2)**	** − 628.4 (0.516)**	**873.2 (0.003)**
Hospitalizations	1,519.6 (6,178.4)	1,672.7 (6,674.0)	153.1 (0.257)	307.8 (0.083)
Specialist consultations	280.1 (914.7)	423.9 (1,224.5)	143.8 (< 0.001)	165.2 (< 0.001)
Other health professional consultations	1,217.3 (3,177.2)	1,970.5 (10,301.8)	753.2 (0.204)	250.4 (0.047)
Tests	441.3 (660.4)	446.8 (665.9)	5.5 (0.883)	49.0 (0.466)
Use of symptomatic drugs and supplements	518.3 (1,198.6)	743.4 (1,685.0)	225.1 (< 0.001)	200.7 (< 0.001)
Disease-modifying treatments	8,538.4 (7,657.4)	6,895.3 (7,606.8)	− 1643.1 (< 0.001)	147.0 (0.753)
***Non-healthcare costs***	**3348.7 (8087.7)**	**5039.8 (11796.3)**	**1691.1 (0.010)**	**818.8 (0.412)**
°Investments	342.8 (1,243.6)	899.5 (4,095.6)	556.7 (0.017)	478.5 (0.215)
Transportation	1,477.8 (4,214.7)	1,242.2 (2,379.4)	− 235.6 (0.578)	− 205.3 (0.663)
Services (personal assistant and home help)	341.4 (1,879.9)	1,478.9 (6,952.2)	1,137.5 (< 0.001)	1,063.2 (< 0.001)
Informal care	1,186.7 (5,444.4)	1,419.3 (6,442.4)	232.6 (0.339)	292.7 (0.297)
***Patients’ productivity losses***	**7207.8 (11505.2)**	**8202.3 (11989.7)**	**994.5 (0.262)**	**333.4 (0.046)**
**Total costs**	**23337.4 (21423.2)**	**25394.7 (24246.4)**	**2057.2 (0.400)**	**3106.9 (< 0.001)**

°Investments included equipment, aids, and modifications. *SD*, standard deviation; *Coef*, coefficient. The models using clustered sandwich estimator of the variance to take account of the nested effect of living in two geographical areas (i.e., Pavia and Genoa) where the participating MS centers are located. *Coefficient adjusted for age, sex, education level, and EDSS score

Comorbidity increased some healthcare costs (specialist consultations, consultations with other healthcare professionals, use of symptomatic drugs and supplements), as well as non-healthcare costs incurred for services. Details are provided in Table 3.

**Fig. 3:**
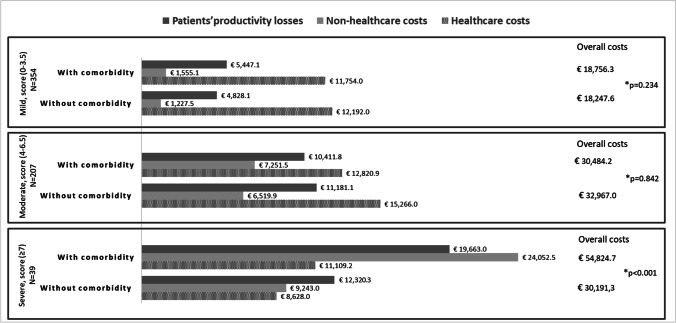
Mean annual cost per patient by presence/absence of comorbidity and disability levels (2020). *The *p* value refers to adjusted (age, sex, and education level) overall cost differences between two comorbidity groups obtained using GLMs with log-link and gamma distributions. A clustered sandwich estimator of the variance was used to take account of the nested effect of living in two geographical regions (i.e., Pavia and Genoa, where the participating MS centers are located)

Between-group analysis of the annual total costs stratified by disability (mild: EDSS 0–3; moderate: EDSS 4–6.5; severe: EDSS ≥ 7) showed a significant mean adjusted incremental cost in the patients with severe disability (€21,853.1, *p* < 0.001) (Fig. 3).

**Fig. 4:**
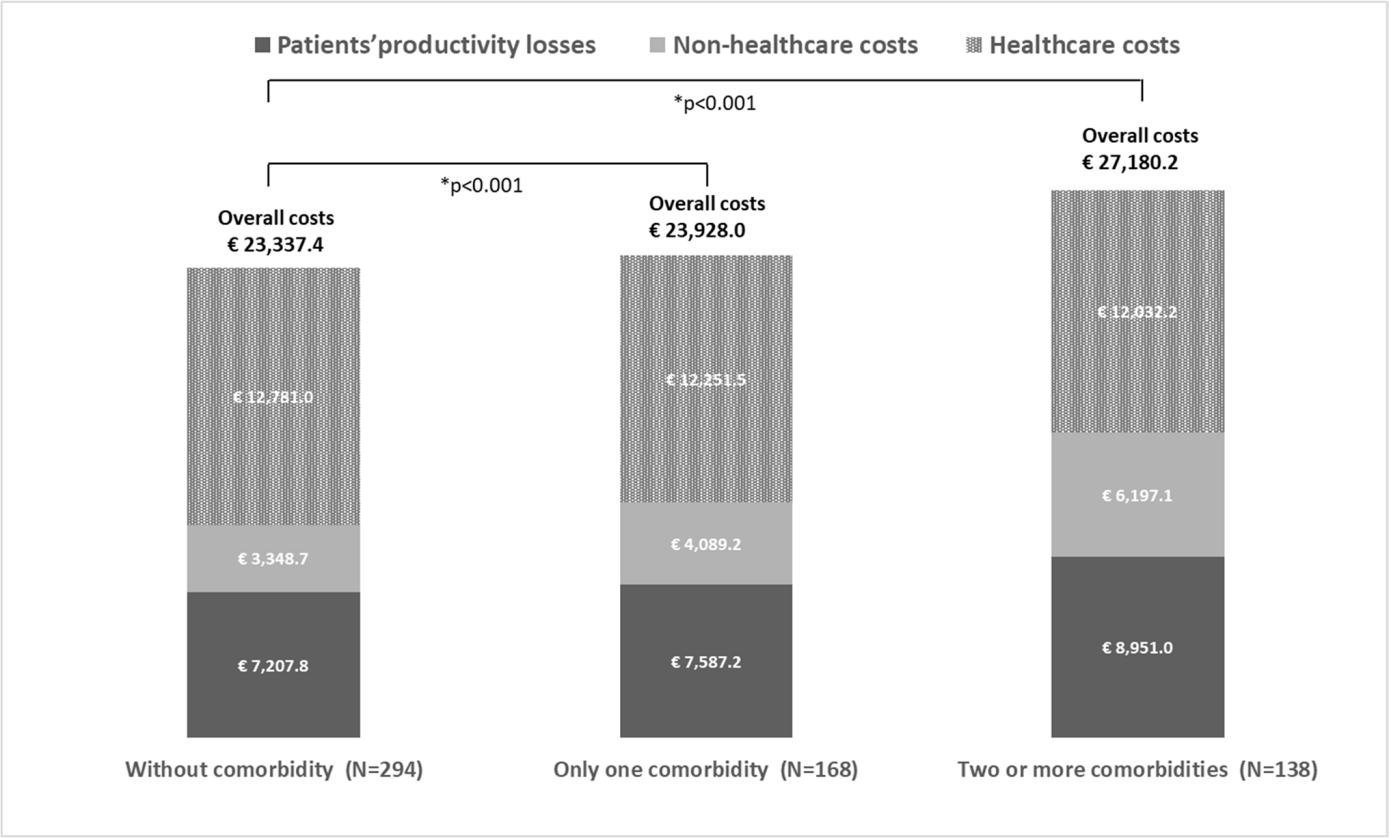
Mean annual cost per patient by number of comorbidities (2020). *The *p* value refers to adjusted (age, sex, education level, and EDSS score) overall cost differences by number of comorbidities (without *vs* only one comorbidity and without *vs* two or more comorbidities) obtained using GLMs with log-link and gamma distributions. A clustered sandwich estimator of the variance was used to take account of the nested effect of living in two geographical regions (i.e., Pavia and Genoa, where the participating MS centers are located)

Moreover, the overall annual costs increased with an increasing number of comorbid conditions (Fig. 4).

Compared with the pwMS without comorbidity, we observed overall adjusted cost differences of €1,679.6 in pwMS with one comorbid condition (*p* < 0.001) and €4,980.8 in those with two or more comorbid conditions (*p* < 0.001).

Finally, the conditions associated with the greatest significant adjusted incremental total costs were depression (€9,070.9, *p* < 0.001), anxiety (€4,248.4, *p* = 0.035), and hypertension (€4,860.4, *p* < 0.001).

## Discussion

We analyzed comorbidity in a large cohort of pwMS, finding 51.0% to have at least one comorbid condition. This result is in line with previous literature reports, in which 40 to 66% of pwMS have at least one additional disease [24, 25]. Of note, 23% of our sample reported having more than one coexisting condition. In the present study, as in the literature, [10, 17, 26] hypertension, depression, and anxiety were the most frequent comorbid conditions in MS. Our data also confirm that comorbidity is associated with low educational level (a socio-economic status proxy) and that the risk of comorbidity increases with age and affects employment status. [27]

This study, which estimates disease costs in pwMS with and without comorbidity, provides evidence on the economic impact of comorbid conditions in this population. The analysis used a regression-based bottom-up approach to estimate disease costs. [28]

PwMS with comorbidity were found to incur higher costs than the group without comorbidity, a result driven largely by their greater use of both healthcare and non-healthcare resources; this group also experienced higher productivity losses. The impact of comorbid conditions on healthcare and non-healthcare resource use in pwMS seems to reflect the challenges inherent in managing coexisting conditions, and shows that these patients need extra support related to their comorbidity. Our results support the findings of other authors, who reported that MS patients with comorbidity have a higher risk of hospitalization and are more likely to have specialist consultations. [29, 30]

We estimated an incremental annual cost per patient of €3,106.9 attributable to comorbid conditions (corresponding to 13% of the total cost), and also showed significant incremental costs due to healthcare resource use and productivity losses, after controlling for potential confounders. Overall, healthcare costs, heavily influenced by DMT costs, accounted for around half of the disease costs. As expected, our results showed that pwMS with comorbidity were less likely to use DMTs (OR = 0.59, *p* = 0.003) and incurred lower costs for these drugs (€6,895.3 vs. €8,538.4, *p* < 0.001), although confounding factors were found to influence these outcome measures. In the case of DMT costs, in particular, we observed a “change in direction” of the incremental cost, after controlling for the potential confounders: unadjusted incremental cost, − 1,643.1 EUR (*p* < 0.001); adjusted incremental cost, + 147.0 EUR (*p* = 0.753). This effect obviously influences the overall healthcare cost trend.

Indeed, subjects with comorbid conditions, being older, with a progressive disease course and moderate/severe disability, are usually less suitable candidates for DMTs. Comorbidity may be a contraindication when prescribing a DMT, and a pre-existing comorbid condition may also influence DMT adherence, persistence, tolerability, and possibly effectiveness. As Zhang and co-workers observed in a large Canadian study, the likelihood of starting DMTs decreased with an increasing number of comorbid conditions. [31]

MS disability exponentially affects annual costs: severe disability was seen to produce an incremental cost of €21,853.1. Moreover, the data also highlighted an additive effect of the presence of more than one comorbidity, showing a relationship between number of comorbidities and increased cost.

Our important finding of increased costs linked to loss of productivity in pwMS with comorbidity confirm that most comorbid conditions with debilitating effects on patients’ lives can reduce work productivity and increase missed work days [32]. As already reported, comorbidity in pwMS is linked to higher sickness absence and disability pension rates [10, 33]. Productivity loss affects the total disease costs and places a major burden on pwMS and their families [13, 18], and our results confirm that the presence of comorbidity exacerbates this. Finally, we examined the impact of the presence of different specific associated conditions on the economic burden of MS; due to the low frequency of some of them, we analyzed only those reported with a frequency > 5%. Depression, reported by 15.7% of the subjects with comorbidity, was associated with the largest adjusted incremental cost, i.e., €9,070.9 (making these patients’ economic burden around 40% greater than that of pwMS without comorbidity). This result suggests that early management of depression could be important, given its potentially considerable clinical and socio-economic repercussions.

This study has certain limitations, first of all in relation to the source of the comorbidity data used, which in this research, was obtained from self-report instruments. While it should be noted that no comorbidity data source can be considered the gold standard in every circumstance, the validity and reliability of self-reported comorbidity data in MS varies by condition. Accuracy is high for chronic conditions that are well defined, require ongoing care, or cause disability. The self-report approach is less accurate for conditions, such as arthritis, where diagnostic criteria are less precise, and may vary in accuracy according to socio-demographic characteristics [34]. Second, the study design was not able to discriminate between comorbid conditions and complications or symptoms. It can sometimes be challenging to decide whether conditions such as depression and anxiety, for example, are actually complications or symptoms of MS. We here chose to treat them as comorbid conditions rather than symptoms of MS because in some individuals they appeared to occur independently of the MS. Third, in Italy, an individual receiving care is generally reimbursed for all medical treatment on the basis of rates established by the National Healthcare System (NHS), unlike practices in other countries. Access to care in Italy is very easy, and both the physician and the patient are allowed considerable decisional autonomy, and can adapt treatments as they wish [35]. However, it is necessary to point out the inequitable distribution of NHS services across geographic areas of Italy (better in the north than the south) [36]. The same consideration has to be made as regard of Italian social security systems that provide welfare and economic benefits such as prosthetic supply, vehicle adaptations, tax relief depending on the different disability levels [37]. Therefore, a major limitation of our results concerns their generalizability to Italian geographic areas with less access to services or to other countries with different healthcare systems.

The major strength of the study was that it evaluated the economic impact of comorbidity in a sample of pwMS considering not only healthcare, but also non-healthcare costs and the cost of lost productivity. In addition, estimates based on a bottom-up approach are more accurate because the method manages to capture all costs more effectively. [28]

In conclusion, comorbidities increase the complexity of patient management and have health, social, and economic consequences for pwMS. Our findings, providing an exhaustive picture of the total economic burden of illness in this population, suggest that comorbidity deserves to be taken properly into account, from an economic as well as a clinical perspective, in treatment and management plans drawn up for patients with MS.
